# Increased Cerebrospinal Fluid Lactate Levels in Schizophrenia and Major Depressive Disorder: NCNP Biobank Study in Japan

**DOI:** 10.1002/npr2.70096

**Published:** 2026-03-03

**Authors:** Hideo Hagihara, Kotaro Hattori, Hiroshi Kunugi, Tsuyoshi Miyakawa

**Affiliations:** ^1^ Division of Systems Medical Science, Center for Medical Science Fujita Health University Toyoake Aichi Japan; ^2^ Department of Bioresources Medical Genome Center, National Centre of Neurology and Psychiatry Tokyo Japan; ^3^ Department of Mental Disorder Research National Institute of Neuroscience, National Centre of Neurology and Psychiatry Tokyo Japan; ^4^ Department of Psychiatry Teikyo University School of Medicine Tokyo Japan

**Keywords:** cerebrospinal fluid, major depressive disorder, metabolism, neural hyperexcitation, schizophrenia

## Abstract

**Aim:**

Altered brain energy metabolism related to neural hyperexcitation, which leads to increased lactate levels in the brain and cerebrospinal fluid (CSF), has been implicated in several neuropsychiatric disorders. This study aimed to investigate CSF levels of lactate and related metabolites in Japanese individuals with schizophrenia and major depressive disorder (MDD), using samples from the National Center for Neurology and Psychiatry biobank.

**Methods:**

CSF levels of lactate, pyruvate, and glucose were measured in 27 patients with schizophrenia, 26 patients with MDD, and 27 age‐matched non‐psychiatric controls. Analyses were conducted by diagnostic groups and demographic variables.

**Results:**

CSF lactate levels were significantly higher in individuals with schizophrenia and MDD compared with controls. CSF glucose levels were significantly elevated in individuals with MDD compared with controls. Pyruvate levels showed no significant group differences. Across all individuals, CSF lactate, pyruvate, and glucose levels were positively correlated. Lactate and glucose levels showed positive correlations with age. No significant associations were found between the three metabolites' levels and medication dosages, except for an effect of imipramine on glucose levels.

**Conclusion:**

This study confirmed elevated CSF lactate levels in Japanese individuals with schizophrenia and MDD, consistent with findings in other populations. The elevation of CSF lactate is unlikely to reflect medication effects and instead may represent an underlying pathophysiology associated with altered brain energy metabolism in the brain.

## Abstract 2

This study demonstrated increased cerebrospinal fluid lactate levels in Japanese patients with schizophrenia and major depressive disorder, consistent with reports from other populations. This elevation is unlikely to be primarily driven by medication and may instead reflect intrinsic alterations in brain energy metabolism.

## Graphical abstract

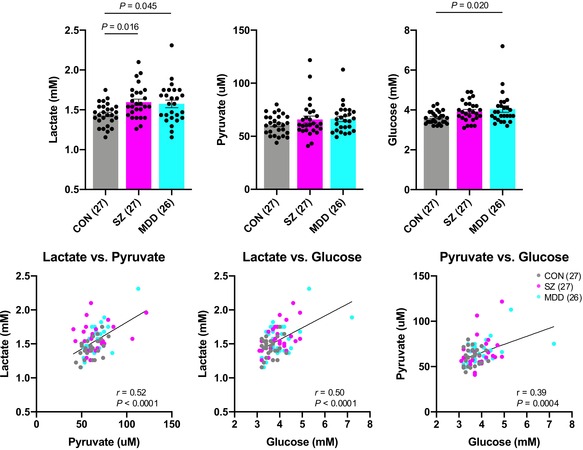

## 1 Introduction

Schizophrenia and major depressive disorder (MDD) are among the most common and severe mental illnesses, sharing several clinical features, including mood disturbances and cognitive impairments. A growing body of evidence suggests that these disorders also share common brain pathophysiology, with neural hyperexcitation and altered energy metabolism increasingly implicated. Studies from our laboratory and others have reported elevated lactate levels and associated decrease in pH, potentially due to neural hyperexcitation, in the brains of patients with schizophrenia [1, 2, 3], MDD [4], and other neuropsychiatric disorders [5]. Furthermore, we recently demonstrated that similar alterations are consistently observed in the brains of various animal models of neuropsychiatric disorders, including models of schizophrenia and MDD [6]. Given that animal models are free from potential confounding factors inherent in human studies, such as the effects of medication and agonal state, these findings support the notion that increased brain lactate levels and decreased pH reflect underlying pathophysiology rather than mere artifacts [2, 6].

Direct brain biopsy in living individuals is ethically infeasible, and although magnetic resonance spectroscopy (MRS) can measure brain lactate in vivo [7, 8], its utility is often limited by detection sensitivity [9, 10]. Cerebrospinal fluid (CSF), while somewhat invasive to collect, serves as a valuable surrogate for assessing the brain's biochemical milieu. Case–control studies examining CSF lactate in schizophrenia remain limited [11, 12]. In the United States, a lumbar CSF study reported elevated lactate in individuals with schizophrenia [13]. In contrast, a study of CSF samples collected in Germany found that drug‐free, first‐onset schizophrenia patients exhibited lower CSF lactate but higher CSF glucose compared with healthy controls [14]. In MDD, ventricular CSF studies using MRS conducted in the United States have yielded mixed findings: one study reported elevated CSF lactate in adolescents with MDD [7], whereas another found no significant difference between adults with MDD and healthy controls [15]. However, to our knowledge, case–control studies in Japanese populations that have systematically examined CSF lactate and related metabolites as primary outcomes in schizophrenia and MDD remain scarce. To address this gap, the present study analyzed CSF samples from Japanese individuals with schizophrenia, MDD, and healthy controls, provided by the National Center for Neurology and Psychiatry (NCNP) Biobank in Japan, to measure lactate and related metabolite levels.

## 2 Materials and Methods

### 2.1 Participants

Participants were recruited from the National Center Hospital, NCNP in Kodaira, Tokyo, Japan. This study included 27 individuals with schizophrenia, 26 individuals with MDD, and 27 healthy controls, matched for age and sex (Table S1). The sample size was determined based on the number of CSF samples that were available for distribution at the time of application from the NCNP Biobank, considering the limited volume of CSF. Most of the participants were of Japanese descent, while a few had close relatives (within three degrees of kinship) of Chinese or Korean nationality. Therefore, the samples analyzed in this study can be broadly characterized as derived from East Asians residing in Japan.

### 2.2 Clinical Assessments

Psychiatric diagnoses were established by qualified psychiatrists using the Japanese version of the Mini‐International Neuropsychiatric Interview (M.I.N.I.) [16, 17], and confirmed according to the criteria of the Diagnostic and Statistical Manual of Mental Disorders, Fourth Edition (DSM‐IV) [18], based on M.I.N.I. results, unstructured interviews, and available medical records. Healthy controls had no history of psychiatric service contact. Exclusion criteria included a history of central nervous system disease, severe head injury, substance abuse, or intellectual disability. CSF metabolite levels may be influenced by various physiological and clinical factors, including seizure activity [19, 20, 21], hypertension [22], systemic glycemic status such as diabetes mellitus [23], time of CSF collection [24, 25], interval since the last meal [24, 26], and medication use [13]. Information regarding these variables, as well as the presence of hyperlipidemia, bronchial asthma, and allergy, was recorded when available. Medication dosages were standardized by calculating total chlorpromazine and imipramine equivalents according to published guidelines [27].

### 2.3 CSF Sample Collection

Collection, storage, and distribution of human samples and associated data were supported by the NCNP Biobank, a member of National Center Biobank Network (NCBN). Following written informed consent, participants underwent a lumbar puncture at the L3–L4 or L4–L5 interspace under local anesthesia, as previously described [28]. The samples were stored at −80°C until analysis.

### 2.4 Measurements of Lactate, Pyruvate, and Glucose Concentrations

CSF lactate, pyruvate, and glucose concentrations were measured as previously described [2]. Briefly, lactate concentrations were determined using a multi‐assay analyzer (GM7 MicroStat; Analox Instruments, London, UK) and a lactate reagent (GMRD‐103, Analox Instruments) in accordance with the manufacturer's instructions. Preliminary tests confirmed a linear, volume‐dependent increase in measured lactate values using 5, 10, and 20 μL of CSF samples (*r* ^2^ > 0.99). Based on this linearity, 10 μL of sample was used for all lactate measurements. Glucose concentrations were measured using the same analyzer with a glucose reagent (GMRD‐002A, Analox Instruments), analyzing 10 μL of sample. Pyruvate concentrations were determined using a pyruvate assay kit (BioVision, Mountain View, CA). Preliminary tests using 1, 5, and 10 μL of CSF confirmed linear, dose‐dependent increases in measured pyruvate values (*r* ^2^ > 0.99). Accordingly, 5 μL of sample was used for all pyruvate measurements. Fluorescence intensities were quantified using a microplate reader equipped with a spectrofluorometer (ARVO X, PerkinElmer).

### 2.5 Statistical Analysis

Statistical analyses were performed to examine (1) group differences in CSF metabolite levels and demographic/clinical variables, (2) associations between metabolite concentrations and demographic or clinical variables, and (3) the diagnostic performance of each metabolite.

#### 2.5.1 Group Comparisons

Differences in CSF lactate, pyruvate, and glucose levels among the three diagnostic groups (schizophrenia, MDD, and controls) were assessed using one‐way analysis of variance (ANOVA), followed by Tukey's post hoc test to identify pairwise differences while controlling for multiple comparisons. For two‐group comparisons, unpaired *t*‐tests were applied. To evaluate group differences in CSF collection time and interval since the last meal, the nonparametric Kruskal–Wallis test was used, as it appropriately accounts for the nonlinear characteristics inherent to circular time variables. These statistical procedures were selected because the primary objective was to test mean differences in continuous variables across independent groups. Categorical variables, such as sex distribution, were analyzed using chi‐squared test. Analyses were conducted using GraphPad Prism (version 8.4.2; GraphPad Software, San Diego, CA).

#### 2.5.2 Correlation and Association Analyses

Pearson correlation tests were performed to examine linear associations between metabolite levels and continuous variables, including age, duration of illness, and medication dosage. To account for potential confounding factors, multiple linear regression (MLR) models were constructed with metabolite concentrations as dependent variables and diagnosis, age, and medication doses (chlorpromazine or imipramine equivalents) as independent variables. MLR was selected to determine whether diagnostic effects remained significant after statistically controlling for known covariates that may influence CSF metabolite levels. Analyses were performed using GraphPad Prism. Analysis of covariance (ANCOVA) was conducted using IBM SPSS Statistics version 29.0.1.0 (IBM Corp., Armonk, NY, USA). In the ANCOVA, pairwise comparisons were performed based on estimated marginal means.

#### 2.5.3 Diagnostic Performance Analyses

Receiver operating characteristic (ROC) curve analyses were conducted using logistic regression models to evaluate the ability of each metabolite to discriminate patients from controls. Leave‐one‐out cross‐validation (LOOCV) was applied to obtain unbiased estimates of model performance, as it maximizes data use in small samples and reduces overfitting. All metabolite values were standardized (*z*‐scored) prior to analysis to eliminate differences in scale and units. Permutation importance analysis was employed to assess the relative contribution of each metabolite to model performance: each predictor was randomly permuted while others were held constant, and the resulting decrease in the area under the curve (AUC) was interpreted as its importance. Analyses were performed using RStudio (version 2025.05.0 + 496) with the caret, pROC, and dplyr packages.

### 2.6 Power Analysis

A post hoc power analysis was conducted to evaluate whether the available sample size provided adequate statistical power to detect group differences in CSF metabolite levels. Using the observed effect sizes from the ANOVA models, statistical power (1–β error probability) was calculated for each metabolite. Analyses were performed using G*Power (version 3.1). Detailed results of the power analysis are provided in Table S2.

## 3 Results

### 3.1 Clinical Characteristics of Participants

Table S1 summarizes the demographic and clinical characteristics of the participants. The three groups did not differ significantly in age (controls: 40.4 ± 7.8 years, schizophrenia: 40.1 ± 9.9 years, MDD: 40.4 ± 8.3 years; all values are presented as mean ± standard deviation; Bartlett's test: *p* = 0.42; one‐way ANOVA: *p* = 0.99) or sex ratio (controls: 13 males/14 females; schizophrenia: 14 males/13 females; MDD: 13 males/13 females; chi‐squared test: *χ* ^2^ = 0.074, *p* = 0.96). The duration of illness was 14.8 ± 9.7 years in schizophrenia and 8.5 ± 5.7 years in MDD. Chlorpromazine equivalent doses were 651.7 ± 1216.0 mg in schizophrenia and 14.0 ± 28.9 mg in MDD, and imipramine equivalent doses were 5.6 ± 20.0 mg in schizophrenia and 131.9 ± 150 mg in MDD. The time of CSF collection ranged from 10:00 to 15:01 (median 13:34) in schizophrenia, 10:00 to 16:30 (median 13:47) in MDD, and 10:00 to 14:30 (median 11:00) in controls. Sampling time differed significantly across diagnostic groups (*p* < 0.004, Kruskal–Wallis test). The interval since the last meal ranged from 0.5 to 19.0 h (median 2.0 h) in schizophrenia, 0.5 to 14.0 h (median 3.0 h) in MDD, and 0.5 to 14.0 h (median 3.0 h) in controls, with no significant group difference (*p* = 0.52, Kruskal–Wallis test).

### 3.2 Increased CSF Lactate Levels in Schizophrenia and MDD

**FIGURE 1:**
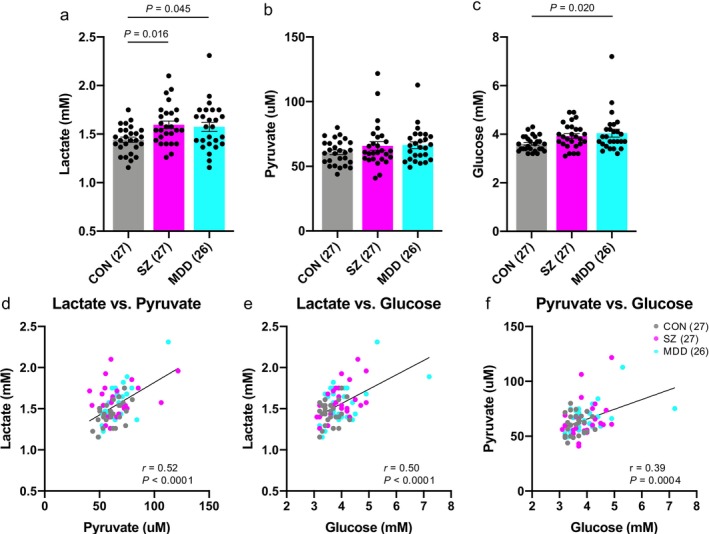
Increased CSF lactate levels in patients with schizophrenia and MDD. (a–c) Bar graphs showing lactate (a), pyruvate (b), and glucose levels (c) in the CSF of control individuals (gray), patients with schizophrenia (SZ) (magenta), and patients with MDD (turquoise). Each dot represents an individual. P‐values were calculated using Tukey's multiple comparison test following one‐way ANOVA. (d–f) Scatter plots showing correlations between lactate and pyruvate levels (d), lactate and glucose levels (e), and pyruvate and glucose levels (f). *r* indicates the Pearson correlation coefficient. Error bars represent the standard error of the mean (SEM).

One‐way ANOVA (*F*_2,77_ = 4.74, *p* = 0.012), followed by Tukey's multiple comparison test, revealed significantly higher CSF lactate levels in individuals with schizophrenia (*p* = 0.016) and MDD (*p* = 0.045) compared with healthy controls (Figure 1a). CSF pyruvate levels showed no significant group differences (*F*_2,77_ = 1.32, *p* = 0.27, One‐way ANOVA; Figure 1b). Glucose levels differed significantly among groups (*F*_2,77_ = 4.11, *p* = 0.020, One‐way ANOVA), with a significant increase observed in MDD (*p* = 0.020), but not in schizophrenia (*p* = 0.11) compared with controls (Figure 1c). Post hoc power analyses suggested that the sample size was sufficient to detect group differences in lactate and glucose, but not in pyruvate (Table S2). Significant positive correlations were observed among the three metabolites across all individuals: lactate and pyruvate (*r* = 0.52, *p* < 0.0001), lactate and glucose (*r* = 0.50, *p* < 0.0001), and pyruvate and glucose (*r* = 0.39, *p* = 0.0004) (Figures 1d–f).

### 3.3 No Significant Effects of Age, Sex, or Medication on Increased CSF Lactate Levels in Disease Conditions

**FIGURE 2:**
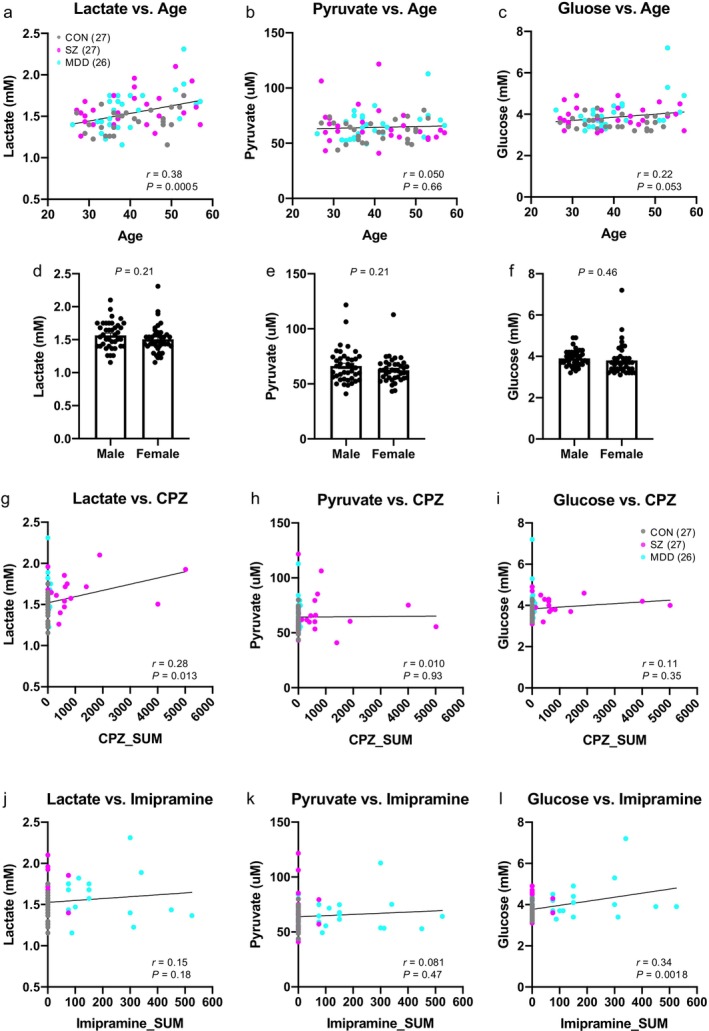
Effects of age, sex, or medication on CSF metabolite levels. (a–c) Scatter plots showing correlations between CSF metabolite levels and ages (a, lactate; b, pyruvate; c, glucose). *r* indicates the Pearson correlation coefficient. (d–f) Bar graphs of lactate (a), pyruvate (b), and glucose levels (c) by sex across all individuals. *p*‐values were calculated using unpaired *t*‐test. Error bars represent SEM. (g–i) Scatter plots showing correlations between CSF metabolite levels and chlorpromazine (CPZ) equivalent doses (g, lactate; h, pyruvate; i, glucose). (j–l) Scatter plots showing correlations between CSF metabolite levels and imipramine equivalent doses (j, lactate; k, pyruvate; l, glucose). *r* indicates the Pearson correlation coefficient.

CSF lactate levels showed a significant positive correlation with age (*r* = 0.38, *p* = 0.0005; Figure 2a) but no significant difference by sex (*p* = 0.21; Figure 2d). Lactate levels were also positively correlated with chlorpromazine equivalent doses (*r* = 0.28, *p* = 0.013; Figure 2g), but not with imipramine equivalents (*r* = 0.15, *p* = 0.18; Figure 2j). Multiple linear regression (MLR) analysis confirmed that, after controlling for age and chlorpromazine equivalents, diagnosis remained a significant predictor of lactate levels for both schizophrenia (diagnosis: |*t*| = 2.60, *p* = 0.012) and MDD (diagnosis: |*t*| = 2.43, *p* = 0.019).

CSF pyruvate levels showed no significant correlation with age (*r* = 0.050, *p* = 0.66; Figure 2b), sex (*p* = 0.21; Figure 2e), chlorpromazine equivalents (*r* = 0.010, *p* = 0.93; Figure 2h), or imipramine equivalents (*r* = 0.081, *p* = 0.47; Figure 2k), and thus no MLR analysis was conducted for pyruvate.

Glucose levels showed a trend toward correlation with age (*r* = 0.22, *p* = 0.053; Figure 2c) but no significant sex difference (*p* = 0.46; Figure 2f) or correlation with chlorpromazine equivalents (*r* = 0.11, *p* = 0.36; Figure 2i), while they were significantly correlated with imipramine equivalents (*r* = 0.29, *p* = 0.034; Figure 2l). MLR analysis indicated that diagnosis significantly predicted glucose levels for schizophrenia (diagnosis: |*t*| = 2.71, *p* = 0.0092), but not for MDD (diagnosis: |*t*| = 1.01, *p* = 0.32); the effect of imipramine was significant for MDD (|*t*| = 2.40, *p* = 0.020).

### 3.4 Effects of Other Clinical Variables on CSF Metabolite Levels

Given the limited availability of clinical records, subgroup analyses were conducted across all participants for hypertension, diabetes, hyperlipidemia, seizure history, bronchial asthma, and allergy. Higher CSF lactate and pyruvate levels were observed in individuals with hypertension, and higher glucose levels were observed in those with diabetes, hyperlipidemia, or a history of seizures (Figure S1). No clinical variables were associated with lower metabolite levels (Figure S1).

In patient samples, a significant correlation between duration of illness and CSF metabolite levels was observed only for lactate in individuals with MDD, showing a negative correlation (*r* = −0.55, *p* = 0.010; Figure S2).

Because a significant group difference was observed in the time of CSF collection, we performed an ANCOVA including sampling time as a covariate. After adjustment for CSF sampling time, CSF lactate levels remained significantly higher in patients with schizophrenia (mean difference [MD] = 0.16, standard error [SE] = 0.061, *p* = 0.012) and MDD (MD = 0.14, SE = 0.058, *p* = 0.021) compared with controls. No significant group differences were observed for pyruvate (schizophrenia: MD = 1.31, SE = 3.99, *p* = 0.74; MDD: MD = 2.90, SE = 3.85, *p* = 0.45). For glucose, no significant difference was observed in schizophrenia (MD = 0.22, SE = 0.17, *p* = 0.22), whereas a significant increase was detected in MDD compared with controls (MD = 0.35, SE = 0.17, *p* = 0.039). These results were consistent with those obtained from the simple one‐way ANOVA, indicating that the observed group differences were not attributable to differences in CSF sampling time.

### 3.5 Moderate Diagnostic Performance of CSF Lactate Levels for Schizophrenia

ROC curve analysis based on LOOCV and logistic regression was performed to evaluate the discriminative power of each metabolite. LOOCV provides a strict evaluation of generalization performance by iteratively using each sample as a validation case. In schizophrenia, CSF lactate (AUC = 0.69, *p* = 0.0097) and glucose (AUC = 0.66, *p* = 0.023) demonstrated moderate but significant discriminative ability, while pyruvate did not (AUC = 0.48, *p* = 0.61) (Figures S3a, S3c, S3d). The combined model using all three metabolites exhibited the highest AUC but did not significantly improve diagnostic accuracy compared with lactate alone (*p* = 0.39; Figure S3c). In MDD, CSF glucose (AUC = 0.66, *p* = 0.024) showed moderate discriminative ability, whereas lactate (AUC = 0.63, *p* = 0.058) or pyruvate (AUC = 0.54, *p* = 0.29) did not (Figures S3b, S3c, S3d). Combining the three metabolites did not significantly enhance diagnostic performance compared with lactate alone (*p* = 0.12; Figure S3c).

Permutation importance was applied to estimate the relative contribution of each CSF metabolite to diagnostic classification performance. For schizophrenia, lactate showed the strongest contribution, with glucose providing additional support (Figures S3e, S3f). For MDD, glucose was the most influential metabolite, while lactate contributed to a lesser extent (Figures S3e, S3f).

## 4 Discussion

In this study, we demonstrated three major findings: (1) CSF lactate levels were significantly elevated in both schizophrenia and MDD; (2) CSF glucose levels were elevated in MDD, although this increase may be partially influenced by antidepressant treatment; and (3) these alterations remained significant after adjusting for available demographic and clinical covariates. Additionally, CSF metabolites showed moderate diagnostic performance in ROC analyses using LOOCV.

Our observation of elevated CSF lactate levels in individuals with schizophrenia or MDD in the Japanese population is consistent with findings from studies conducted in the USA [7, 8, 13], suggesting that this may be a phenomenon specific to particular countries or regions. Increased CSF lactate has also been reported in other neuropsychiatric disorders, such as Alzheimer's disease (AD) [29, 30] and bipolar disorder [13, 31], suggesting that it is not disease‐specific. Rather, elevated CSF lactate may represent a transdiagnostic endophenotype associated with neural hyperexcitation, a pathophysiological mechanism shared across multiple brain disorders. Indeed, epileptic seizures, which are common comorbidities in AD [32], have been shown to elevate CSF lactate levels [19, 20, 21]. In the present study, only two subjects had a history of seizures, precluding a systematic assessment of the relationship between seizure activity and CSF lactate elevation. Future studies with larger sample sizes are warranted to clarify this potential association.

We also observed that CSF lactate concentrations were higher in older participants. Similar findings have been reported in humans by MR spectroscopy and CSF analyses [33, 34], as well as in aged rodents [35, 36], where higher brain lactate levels may reflect changes in neural excitation, energy metabolism, and mitochondrial function. Conversely, some studies, including ^13^C‐MR spectroscopy, suggested reduced lactate production with aging [37, 38], and others found no clear age‐related changes [39]. These discrepancies may relate to methodological or population differences, but overall our data add preliminary support that lactate homeostasis in the central nervous system is altered with aging.

A recent study investigated a diagnostic model for depression using plasma metabolomic datasets from over 200 000 participants in the UK Biobank [40]. A machine learning model incorporating the identified 20 most important features, including both metabolic (e.g., lactate, pyruvate, glucose) and non‐metabolic (e.g., age, sex, BMI) variables, achieved AUC values of 0.658 for lifetime depression and 0.716 for current depression [40]. In our study, the AUC value reached 0.63 using lactate alone and 0.77 when combining the three metabolites, showing performance comparable to or slightly better than that of the previous large‐scale study. If further validated, these findings suggest that lactate and related metabolites in fluid samples from living individuals may serve as promising predictors of depression.

We also note several limitations to this study. Regarding sample size, a post hoc power analysis showed that the comparisons of lactate and glucose exhibited medium‐to‐large effect sizes (*d* = 0.67–0.88), yielding adequate statistical power (0.78–0.94) with the current sample sizes (Table S2). These results indicate that the study had sufficient sensitivity to detect group differences in lactate and glucose. In contrast, the effect sizes for pyruvate were small (*d* = 0.36–0.48), with low achieved power (0.36–0.53) (Table S2), suggesting that the present sample size may have been insufficient to detect potential group differences in pyruvate. Thus, the null findings for pyruvate should be interpreted with caution, considering the possibility of underpowered detection. Furthermore, with respect to controlling for covariates, several physiological factors, such as seizure activity [19, 20, 21], hypertension [22], diabetes mellitus [23], have been suggested as potential confounders of CSF metabolite levels. However, detailed information on these factors was not available for all participants, and therefore ANCOVA or other covariate‐adjusted analyses were not performed. Importantly, the number of individuals presenting with any of these conditions was very small (2–6 participants across the three groups; Table S1), making it unlikely that these factors substantially influenced the diagnostic effects observed in CSF metabolite levels. Nevertheless, these issues related to sample size and potential confounding factors remain important challenges to be addressed in future studies.

Collectively, our findings support the notion that altered brain energy metabolism, as reflected by elevated CSF lactate, may represent a shared neurobiological pathway underlying a range of neuropsychiatric disorders characterized by neural hyperexcitation.

## Author Contributions

H.H. and T.M. conceived and designed the study and drafted the manuscript. H.H., K.H., and T.M. analyzed the data. H.H. conducted the measurement experiments. K.H. and H.K. collected the samples. All authors reviewed and approved the final manuscript.

## Funding

The collection of CSF samples at the NCNP Biobank was partly supported by grants from an Intramural Research Grant (6–1 and 6–7) for Neurological and Psychiatric Disorders of NCNP. Sample usage was supported by the AMED Biobank Network (AMED Platform Program for Promotion of Genome Medicine [JP21km0405401]). Metabolites measurement experiments were supported by JSPS KAKENHI (Grant Numbers JP20H00522 and JP18K07378), and MEXT Promotion of Distinctive Joint Research Center Program (Grant Number FY2018‐2020 JPMXP0618217663, FY2021‐2023 JPMXP0621467949).

## Ethics Statement

This study was approved by the ethics committees of Fujita Health University (approval no. HM22‐249), Teikyo University (approval no. I‐COI2022‐0274), and the NCNP (approval no. NCNPBB‐0144).

## Consent

All participants provided written informed consent.

## Conflicts of Interest

The authors declare no conflicts of interest. Tsuyoshi Miyakawa is the Editor‐in‐Chief of Neuropsychopharmacology Reports and a co‐author of this article. He was excluded from editorial decision‐making related to the acceptance and publication of this article. Editorial decision‐making was handled independently by Editor‐in‐Chief, Tsuyoshi Miyakawa, to minimize bias.

## Supporting information


**Figure S1:** Effects of other clinical variables on CSF metabolite levels.
**Figure S2:** Association between duration of illness and CSF metabolite levels.
**Figure S3:** Classification performance of CSF metabolites for distinguishing schizophrenia or MDD from controls.

**Table S1:** Donor demographics and raw data analyzed in this study.
**Table S2:** Results of post hoc power analysis.

## Data Availability

The data analyzed in this study are provided in Table S1.

## References

[1] A. E. Dogan , C. Yuksel , F. Du , V.‐A. Chouinard , and D. Öngür , “Brain Lactate and pH in Schizophrenia and Bipolar Disorder: A Systematic Review of Findings From Magnetic Resonance Studies,” Neuropsychopharmacology 43 (2018): 1681–1690. 29581538 10.1038/s41386-018-0041-9 PMC6006165

[2] H. Hagihara , V. S. Catts , Y. Katayama , et al., “Decreased Brain pH as a Shared Endophenotype of Psychiatric Disorders,” Neuropsychopharmacology 43 (2018): 459–468. 28776581 10.1038/npp.2017.167 PMC5770757

[3] B. S. Pruett and J. H. Meador‐Woodruff , “Evidence for Altered Energy Metabolism, Increased Lactate, and Decreased pH in Schizophrenia Brain: A Focused Review and Meta‐Analysis of Human Postmortem and Magnetic Resonance Spectroscopy Studies,” Schizophrenia Research 223 (2020): 29–42. 32958361 10.1016/j.schres.2020.09.003

[4] H. Hagihara and T. Miyakawa , “Postmortem Evidence of Decreased Brain pH in Major Depressive Disorder: A Systematic Review and Meta‐Analysis,” Translational Psychiatry 14 (2024): 460. 39496593 10.1038/s41398-024-03173-7 PMC11535390

[5] H. Hagihara and T. Miyakawa , “Decreased Brain pH Correlated With Progression of Alzheimer Disease Neuropathology: A Systematic Review and Meta‐Analyses of Postmortem Studies,” International Journal of Neuropsychopharmacology 27 (2024): pyae047. 39422361 10.1093/ijnp/pyae047 PMC11511658

[6] H. Hagihara , H. Shoji , S. Hattori , et al., “Large‐Scale Animal Model Study Uncovers Altered Brain pH and Lactate Levels as a Transdiagnostic Endophenotype of Neuropsychiatric Disorders Involving Cognitive Impairment,” eLife 12 (2024): RP89376. 38529532 10.7554/eLife.89376 PMC10965225

[7] K. A. L. Bradley , X. Mao , J. A. C. Case , G. Kang , D. C. Shungu , and V. Gabbay , “Increased Ventricular Cerebrospinal Fluid Lactate in Depressed Adolescents,” European Psychiatry 32 (2016): 1–8. 26802978 10.1016/j.eurpsy.2015.08.009 PMC4831134

[8] D. C. Shungu , N. Weiduschat , J. W. Murrough , et al., “Increased Ventricular Lactate in Chronic Fatigue Syndrome. III. Relationships to Cortical Glutathione and Clinical Symptoms Implicate Oxidative Stress in Disorder Pathophysiology,” NMR in Biomedicine 25 (2012): 1073–1087. 22281935 10.1002/nbm.2772 PMC3896084

[9] R. J. Maddock and M. H. Buonocore , “MR Spectroscopic Studies of the Brain in Psychiatric Disorders,” Current Topics in Behavioral Neurosciences 11 (2012): 199–251. 22294088 10.1007/7854_2011_197

[10] J. Ernst , A. Hock , A. Henning , E. Seifritz , H. Boeker , and S. Grimm , “Increased Pregenual Anterior Cingulate Glucose and Lactate Concentrations in Major Depressive Disorder,” Molecular Psychiatry 22 (2017): 113. 27184123 10.1038/mp.2016.73

[11] X. Chen , Y. Zhang , H. Wang , L. Liu , W. Li , and P. Xie , “The Regulatory Effects of Lactic Acid on Neuropsychiatric Disorders,” Discover Mental Health 2 (2022): 8. 37861858 10.1007/s44192-022-00011-4 PMC10501010

[12] S. Zhang , J. Xia , W. He , et al., “From Energy Metabolism to Mood Regulation: The Rise of Lactate as a Therapeutic Target,” Journal of Advanced Research 80 (2026): 535–554. 40262720 10.1016/j.jare.2025.04.018 PMC12869207

[13] W. T. Regenold , P. Phatak , C. M. Marano , A. Sassan , R. R. Conley , and M. A. Kling , “Elevated Cerebrospinal Fluid Lactate Concentrations in Patients With Bipolar Disorder and Schizophrenia: Implications for the Mitochondrial Dysfunction Hypothesis,” Biological Psychiatry 65 (2009): 489–494. 19103439 10.1016/j.biopsych.2008.11.010 PMC3752997

[14] E. Holmes , T. M. Tsang , J. T.‐J. Huang , et al., “Metabolic Profiling of CSF: Evidence That Early Intervention May Impact on Disease Progression and Outcome in Schizophrenia,” PLoS Medicine 3 (2006): e327. 16933966 10.1371/journal.pmed.0030327 PMC1551919

[15] J. W. Murrough , X. Mao , K. A. Collins , et al., “Increased Ventricular Lactate in Chronic Fatigue Syndrome Measured by 1H MRS Imaging at 3.0 T. II: Comparison With Major Depressive Disorder,” NMR in Biomedicine 23 (2010): 643–650. 20661876 10.1002/nbm.1512

[16] D. V. Sheehan , Y. Lecrubier , K. H. Sheehan , et al., “The Mini‐International Neuropsychiatric Interview (M.I.N.I.): The Development and Validation of a Structured Diagnostic Psychiatric Interview for DSM‐IV and ICD‐10,” Journal of Clinical Psychiatry 59, no. 20 (1998): 22–33; quiz 34–57. 9881538

[17] T. Otsubo , K. Tanaka , R. Koda , et al., “Reliability and Validity of Japanese Version of the Mini‐International Neuropsychiatric Interview,” Psychiatry and Clinical Neurosciences 59 (2005): 517–526. 16194252 10.1111/j.1440-1819.2005.01408.x

[18] American Psychiatric Association , ed., Diagnostic and Statistical Manual of Mental Disorders: DSM‐IV ; Includes ICD‐9‐CM Codes Effective 1. Oct. 96, American Psychiatric Association; 4th ed. (1994): 886.

[19] M. Süße , K. Gag , L. Hamann , M. J. Hannich , and F. von Podewils , “Time Dependency of CSF Cell Count, Lactate and Blood‐CSF Barrier Dysfunction After Epileptic Seizures and Status Epilepticus,” Seizure 95 (2022): 11–16. 34954628 10.1016/j.seizure.2021.12.007

[20] H. Tumani , C. Jobs , J. Brettschneider , A. C. Hoppner , F. Kerling , and S. Fauser , “Effect of Epileptic Seizures on the Cerebrospinal Fluid – A Systematic Retrospective Analysis,” Epilepsy Research 114 (2015): 23–31. 26088882 10.1016/j.eplepsyres.2015.04.004

[21] A. Chatzikonstantinou , A. D. Ebert , and M. G. Hennerici , “Cerebrospinal Fluid Findings After Epileptic Seizures,” Epileptic Disorders 17 (2015): 453–459. 26575850 10.1684/epd.2015.0779

[22] M. Fujishima , Y. Nakatomi , K. Tamaki , T. Ishitsuka , T. Kawasaki , and T. Omae , “Cerebrospinal Fluid Lactate and Pyruvate Concentrations in Patients With Malignant Hypertension,” Journal of Neurology 231 (1984): 71–74. 6737011 10.1007/BF00313719

[23] H. Yao , S. Sadoshima , Y. Nishimura , et al., “Cerebrospinal Fluid Lactate in Patients With Diabetes Mellitus and Hypoglycaemic Coma,” Journal of Neurology, Neurosurgery, and Psychiatry 52 (1989): 372–375. 2926423 10.1136/jnnp.52.3.372 PMC1032413

[24] M. M. Verbeek , W. G. Leen , M. A. Willemsen , D. Slats , and J. A. Claassen , “Hourly Analysis of Cerebrospinal Fluid Glucose Shows Large Diurnal Fluctuations,” Journal of Cerebral Blood Flow and Metabolism 36 (2016): 899–902. 26945018 10.1177/0271678X16637612 PMC4853846

[25] J. P. Koleske , S. Pan , T. Meehan , et al., “Time‐Of‐Day and Age‐Related Patterns in Cerebrospinal Fluid Glucose and Protein,” Fluids and Barriers of the CNS 22 (2025): 102. 41102834 10.1186/s12987-025-00686-1 PMC12532875

[26] K. Saito , K. Hattori , T. Andou , et al., “Characterization of Postprandial Effects on CSF Metabolomics: A Pilot Study With Parallel Comparison to Plasma,” Metabolites 10 (2020): 185. 32384774 10.3390/metabo10050185 PMC7281358

[27] T. Inada and A. Inagaki , “Psychotropic Dose Equivalence in Japan,” Psychiatry and Clinical Neurosciences 69 (2015): 440–447. 25601291 10.1111/pcn.12275

[28] K. Hattori , M. Ota , D. Sasayama , et al., “Increased Cerebrospinal Fluid Fibrinogen in Major Depressive Disorder,” Scientific Reports 5 (2015): 11412. 26081315 10.1038/srep11412 PMC4469953

[29] C. Liguori , A. Stefani , G. Sancesario , G. M. Sancesario , M. G. Marciani , and M. Pierantozzi , “CSF Lactate Levels, τ Proteins, Cognitive Decline: A Dynamic Relationship in Alzheimer's Disease,” Journal of Neurology, Neurosurgery, and Psychiatry 86 (2015): 655–659. 25121572 10.1136/jnnp-2014-308577

[30] C. Liguori , A. Chiaravalloti , G. Sancesario , et al., “Cerebrospinal Fluid Lactate Levels and Brain [18F]FDG PET Hypometabolism Within the Default Mode Network in Alzheimer's Disease,” European Journal of Nuclear Medicine and Molecular Imaging 43 (2016): 2040–2049. 27221635 10.1007/s00259-016-3417-2

[31] N. Yoshimi , T. Futamura , S. E. Bergen , et al., “Cerebrospinal Fluid Metabolomics Identifies a Key Role of Isocitrate Dehydrogenase in Bipolar Disorder: Evidence in Support of Mitochondrial Dysfunction Hypothesis,” Molecular Psychiatry 21 (2016): 1504–1510. 26782057 10.1038/mp.2015.217 PMC5078854

[32] Y. Xu , L. Lavrencic , K. Radford , et al., “Systematic Review of Coexistent Epileptic Seizures and Alzheimer's Disease: Incidence and Prevalence,” Journal of the American Geriatrics Society 69 (2021): 2011–2020. 33740274 10.1111/jgs.17101

[33] P. T. Zebhauser , A. Berthele , O. Goldhardt , et al., “Cerebrospinal Fluid Lactate Levels Along the Alzheimer's Disease Continuum and Associations With Blood‐Brain Barrier Integrity, Age, Cognition, and Biomarkers,” Alzheimer's Research & Therapy 14 (2022): 61. 10.1186/s13195-022-01004-9 PMC9044672 35473756

[34] P. E. Sijens , T. den Heijer , F. E. de Leeuw , et al., “MR Spectroscopy Detection of Lactate and Lipid Signals in the Brains of Healthy Elderly People,” European Radiology 11 (2001): 1495–1501. 11519564 10.1007/s003300100824

[35] J. M. Ross , J. Öberg , S. Brené , et al., “High Brain Lactate Is a Hallmark of Aging and Caused by a Shift in the Lactate Dehydrogenase A/B Ratio,” Proceedings of the National Academy of Sciences of the United States of America 107 (2010): 20087–20092. 21041631 10.1073/pnas.1008189107 PMC2993405

[36] S. Datta and N. Chakrabarti , “Age Related Rise in Lactate and Its Correlation With Lactate Dehydrogenase (LDH) Status in Post‐Mitochondrial Fractions Isolated From Different Regions of Brain in Mice,” Neurochemistry International 118 (2018): 23–33. 29678731 10.1016/j.neuint.2018.04.007

[37] J. M. N. Duarte , K. Q. Do , and R. Gruetter , “Longitudinal Neurochemical Modifications in the Aging Mouse Brain Measured In Vivo by 1H Magnetic Resonance Spectroscopy,” Neurobiology of Aging 35 (2014): 1660–1668. 24560998 10.1016/j.neurobiolaging.2014.01.135

[38] B. Uthayakumar , H. Soliman , N. D. Bragagnolo , et al., “Age‐Associated Change in Pyruvate Metabolism Investigated With Hyperpolarized 13C‐MRI of the Human Brain,” Human Brain Mapping 44 (2023): 4052–4063. 37219519 10.1002/hbm.26329 PMC10258534

[39] C. Y. Lee , H. Soliman , B. J. Geraghty , et al., “Lactate Topography of the Human Brain Using Hyperpolarized 13C‐MRI,” NeuroImage 204 (2020): 116202. 31557546 10.1016/j.neuroimage.2019.116202

[40] S. Ma , X. Xie , Z. Deng , et al., “A Machine Learning Analysis of Big Metabolomics Data for Classifying Depression: Model Development and Validation,” Biological Psychiatry 96 (2024): 44–56. 38142718 10.1016/j.biopsych.2023.12.015

