# Supplementary material for: Increased Cerebrospinal Fluid Lactate Levels in Schizophrenia and Major Depressive Disorder: NCNP Biobank Study in Japan
Source: Neuropsychopharmacol Rep. 2026 Mar 3;46(1):e70096. doi: 10.1002/npr2.70096 (PMC12956547; doi:10.1002/npr2.70096)
Supplement: Supplementary file 1 — Figure S1: Effects of other clinical variables on CSF metabolite levels. Figure S2: Association between duration of illness and CSF metabolite levels. Figure S3: Classification performance of CSF metabolites for distinguishing schizophrenia or MDD from controls. [file NPR2-46-e70096-s001.pdf]

Supplementary Figures

Increased Cerebrospinal Fluid Lactate Levels in Schizophrenia and Major Depressive Disorder: NCNP Biobank Study in Japan

Hideo Hagihara[1], Kotaro Hattori[2,3], Hiroshi Kunugi[3,4], and Tsuyoshi Miyakawa[1]

[1] Division of Systems Medical Science, Center for Medical Science, Fujita Health University, Toyoake, Aichi 470-1192, Japan

[2] Department of Bioresources, Medical Genome Center, National Centre of Neurology and Psychiatry, Kodaira, Tokyo 187-0031, Japan

[3] Department of Mental Disorder Research, National Institute of Neuroscience, National Centre of Neurology and Psychiatry, Kodaira, Tokyo 187-0031, Japan

[4] Department of Psychiatry, Teikyo University School of Medicine, Tokyo 173-8605, Japan

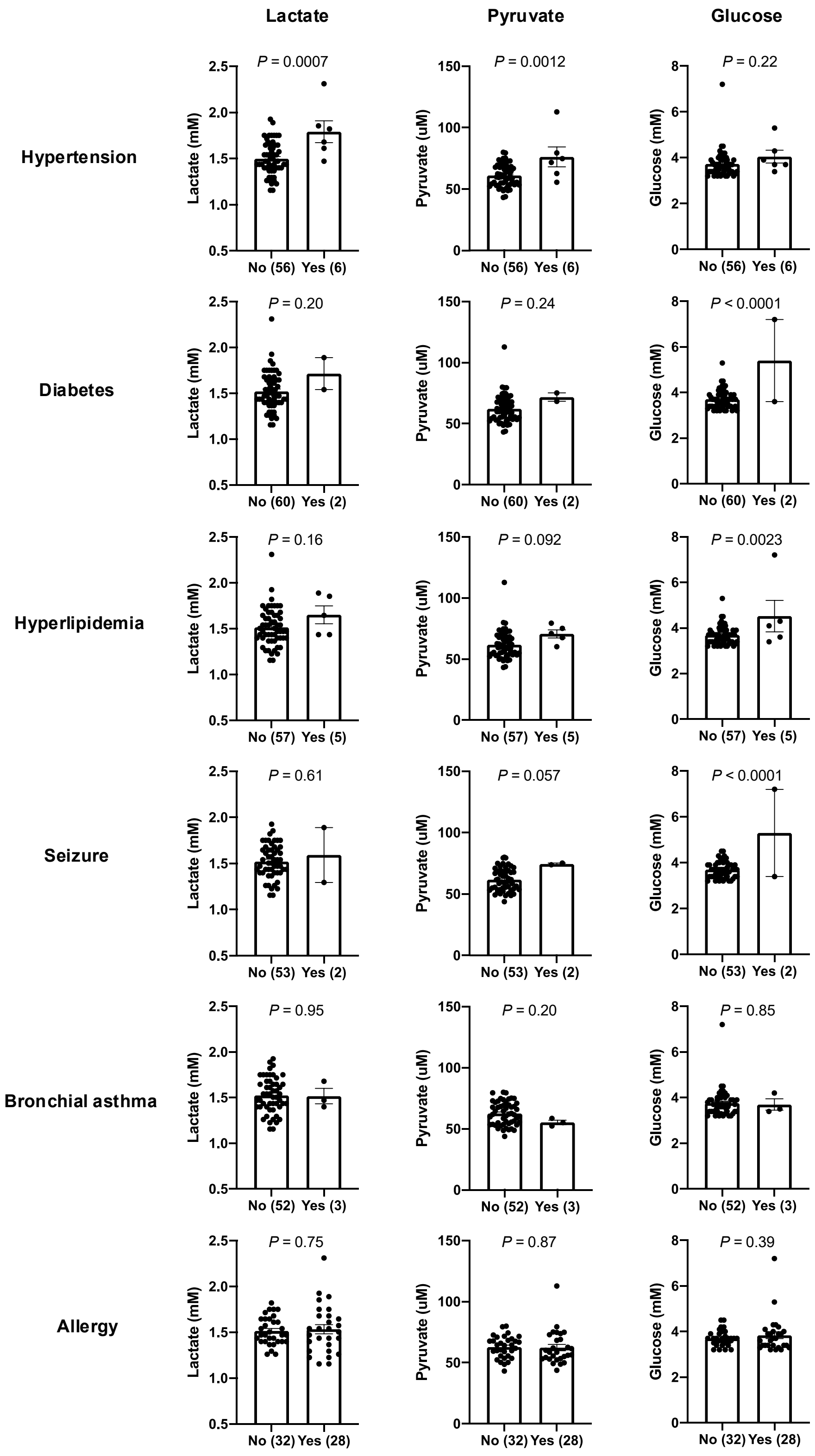
Lactate
Pyruvate
Glucose
Hypertension
Diabetes
Hyperlipidemia
Seizure
Bronchial asthma
Allergy
P = 0.0007
P = 0.0012
P = 0.22
P = 0.20
P = 0.24
P < 0.0001
P = 0.16
P = 0.092
P = 0.0023
P = 0.61
P = 0.057
P < 0.0001
P = 0.95
P = 0.20
P = 0.85
P = 0.75
P = 0.87
P = 0.39
Lactate (mM)
Pyruvate (uM)
Glucose (mM)
No (56) Yes (6)
No (60) Yes (2)
No (57) Yes (5)
No (53) Yes (2)
No (52) Yes (3)
No (32) Yes (28)

Figure S1. Effects of other clinical variables on CSF metabolite levels. Bar graphs showing CSF lactate, pyruvate, and glucose levels in relation to the presence or absence of hypertension, diabetes, hyperlipidemia, seizure history, bronchial asthma, and allergy. P-values were calculated using unpaired t-test. Error bars represent SEM.

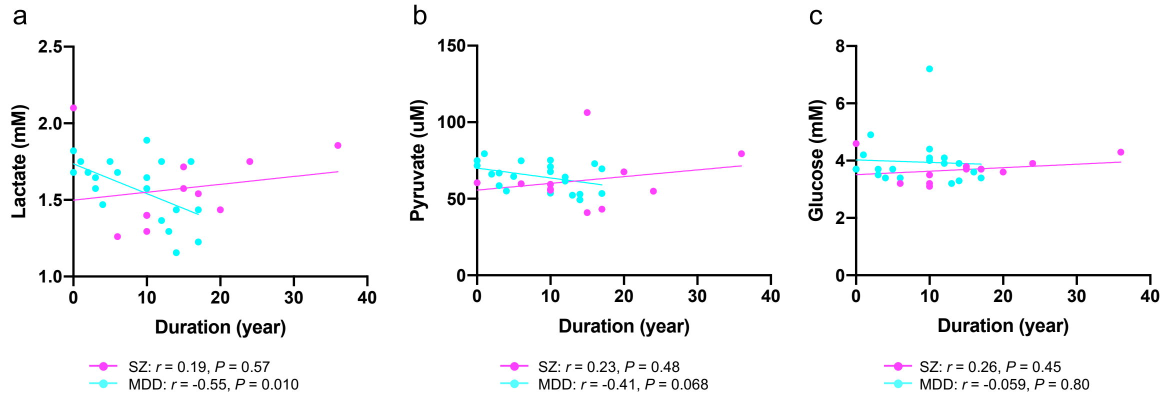

Figure S2. Association between duration of illness and CSF metabolite levels. Scatter plots showing correlations between CSF metabolite levels and duration of illness: (a) lactate, (b) pyruvate, and (c) glucose. *r* indicates the Pearson correlation coefficient. MDD, major depressive disorder; SZ, schizophrenia.

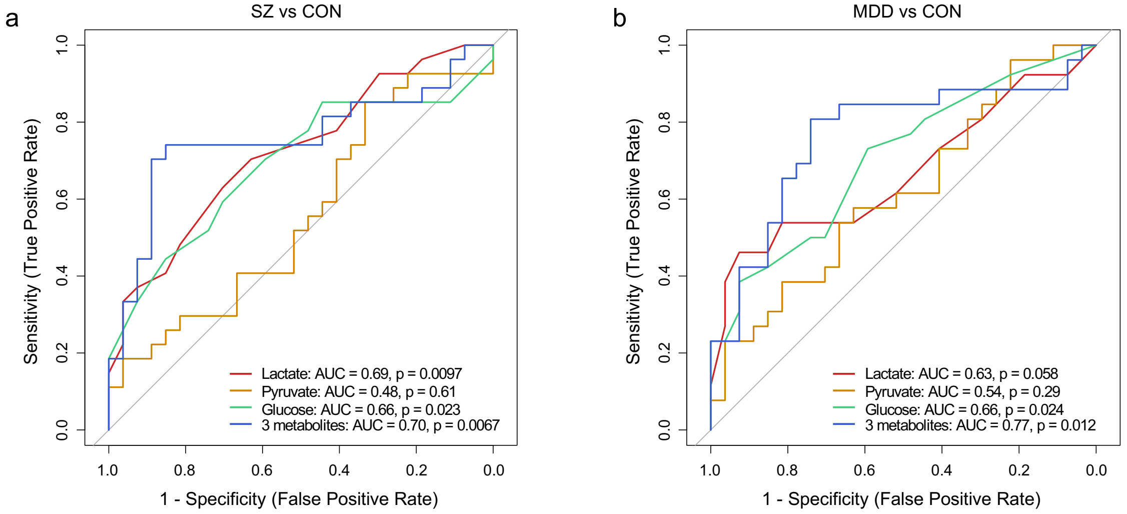

c

Comparison	SZ P-value	MDD P-value
Lactate vs Pyruvate	0.063	0.48
Lactate vs Glucose	0.70	0.70
Lactate vs 3 metabolites	0.39	0.12
Pyruvate vs Glucose	0.18	0.34
Pyruvate vs 3 metabolites	0.029	0.059
Glucose vs 3 metabolites	0.17	0.14

e

Disorder	Metabolite	AUC drop	Relative (%)
SZ	Lactate	0.14	66.4
	Pyruvate	-0.010	-0.3
	Glucose	0.070	33.9
MDD	Lactate	0.105	39.2
	Pyruvate	0.002	0.7
	Glucose	0.161	60.1

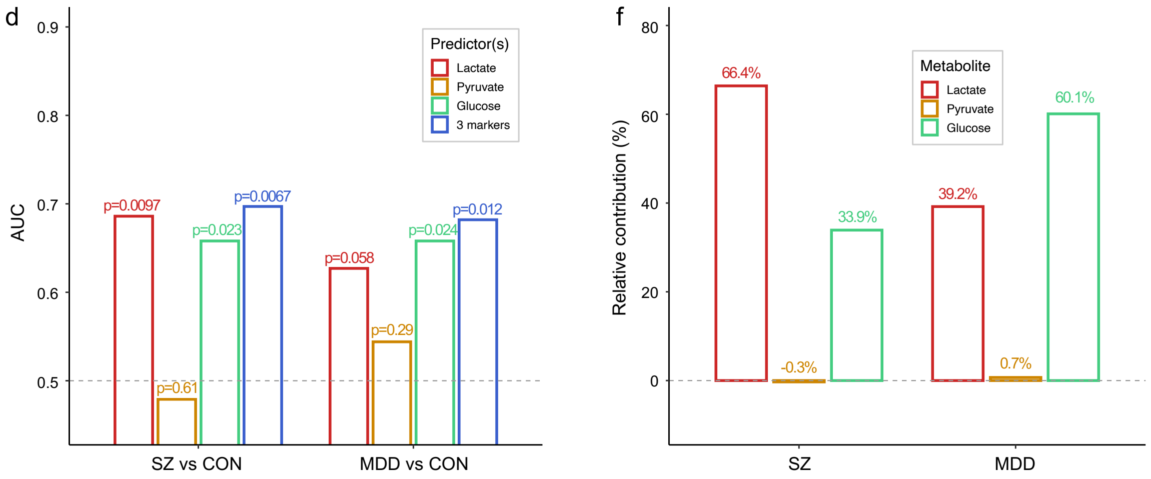

Figure S3. Classification performance of CSF metabolites for distinguishing schizophrenia or MDD from controls. (a, b) Receiver operating characteristic (ROC) curves showing the classification performance of individual and combined CSF metabolic markers (lactate, pyruvate, glucose, and their combination) in distinguishing patients with schizophrenia (SZ) (a) and major depressive disorder (MDD) (b) from controls. p-values were calculated using DeLong's test for the hypothesis that the LOOCV-based

AUC was significantly higher than 0.5 (random classification). **(c)** Comparison of ROC curves (DeLong test). **(d)** Bar graphs summarizing AUC values for each model shown in panels (a) and (c). **(e)** Results of permutation importance analysis, showing absolute (AUC drop) and relative (%) contributions of each metabolite. **(f)** Bar graphs showing the relative contribution of each metabolite to classification performance in permutation importance analysis, based on the decrease in AUC after variable permutation.
